# Valorization of Kiwiberry Leaves Recovered by Ultrasound-Assisted Extraction for Skin Application: A Response Surface Methodology Approach

**DOI:** 10.3390/antiox11040763

**Published:** 2022-04-12

**Authors:** Ana Margarida Silva, Diana Pinto, Manuela M. Moreira, Paulo C. Costa, Cristina Delerue-Matos, Francisca Rodrigues

**Affiliations:** 1 REQUIMTE/LAQV, Instituto Superior de Engenharia do Porto, Rua Dr. António Bernardino de Almeida, 431, 4249-015 Porto, Portugal; ana.silva@graq.isep.ipp.pt (A.M.S.); diana.pinto@graq.isep.ipp.pt (D.P.); manuela.moreira@graq.isep.ipp.pt (M.M.M.); cmm@isep.ipp.pt (C.D.-M.); 2 REQUIMTE/UCIBIO, MedTech-Laboratory of Pharmaceutical Technology, Department of Drug Sciences, Faculty of Pharmacy, University of Porto, Rua de Jorge Viterbo Ferreira, 228, 4050-313 Porto, Portugal; pccosta@ff.up.pt; 3 Associate Laboratory i4HB, Institute for Health and Bioeconomy, Faculty of Pharmacy, University of Porto, 4050-313 Porto, Portugal

**Keywords:** *Actinidia arguta* leaves, response surface methodology, ultrasound-assisted extraction, phenolic compounds, cell viability

## Abstract

This study aims to evaluate the optimal ultrasound-assisted extraction (UAE) conditions of antioxidants polyphenols from *Actinidia arguta* (Siebold & Zucc.) Planch. Ex Miq. (kiwiberry) leaves using a response surface methodology (RSM). The effects of solid:liquid ratio (2.5–10.0% *w*/*v*), time (20–60 min), and intensity (30–70 W/m^2^) on the total phenolic content (TPC) and antioxidant/antiradical activities were investigated. The optimal UAE conditions were achieved using a solid:liquid ratio of 10% (*w*/*v*) and an ultrasonic intensity of 30 W/m^2^ for 31.11 min. The results demonstrated that the optimal extract showed a high TPC (97.50 mg of gallic acid equivalents (GAE)/g dw) and antioxidant/antiradical activity (IC_50_ = 249.46 µg/mL for ABTS assay; IC_50_ = 547.34 µg/mL for DPPH assay; 1440.13 µmol of ferrous sulfate equivalents (FSE)/g dw for ferric reducing antioxidant power (FRAP)) as well as a good capacity to scavenge superoxide and hypochlorous acid (respectively, IC_50_ = 220.13 μg/mL and IC_50_ =10.26 μg/mL), which may be related with the 28 phenolic compounds quantified. The in vitro cell assay demonstrated that the optimal extract did not decrease the keratinocytes’ (HaCaT) viability, while the fibroblasts’ (HFF-1) viability was greater than 70.63% (1000 µg/mL). This study emphasizes the great potential of kiwiberry leaves extracted by UAE for skin application.

## 1. Introduction

The world population is continuously growing and it is estimated that food production will increase by 60% by 2050 [1]. According to the World Health Organization (WHO), the recommended intake of fruits and vegetables should be 400 g per day [2]. Nevertheless, the production of plant-based foods creates high amounts of residues, such as seeds, skins, leaves or pulp, among others [3,4]. The different by-products generated have low added-value and are commonly reused as fertilizers or supplement feeds for animals [3]. However, these by-products are a rich source of different bioactive compounds and their recovery and valorization constitute a major challenge, according to the Sustainable Development Goals of 2030 Agenda [5].

The cultivation of *Actinidia arguta* (Siebold & Zucc.) Planch. ex Miq. has increased in recent years, leading to huge amounts of by-products such as pomace, skins or leaves. The different by-products from Actinidia genus have been widely studied regarding their nutritional and healthy properties [6,7,8,9,10,11,12,13]. Our research group has focused on the valorization of *A. arguta* leaves that are removed in large amounts during production to promote a better fruit maturation [7,9,14]. *A. arguta* leaves present high levels of bioactive compounds with human health benefits, particularly antioxidant and anti-inflammatory compounds [7,8,9,10]. Among them, phenolic compounds are of particular importance, being responsible for the scavenge capacity of reactive species. The free radicals may interact with other molecules, increasing the oxidative stress and, consequently, led to the appearance of several diseases, such as cancer, cardiovascular, neurodegenerative or chronic disorders [15]. In addition, different extracts of *A. arguta* leaves demonstrated a protector effect on intestinal and skin cell lines [7,8,9,10]. Besides that, the reuse and valorization of this by-product may have a positive impact on the environment and socioeconomic activities [16].

The extraction process is an essential step to recover high amounts of bioactive compounds from natural matrices [17,18]. Over the last few years, different green extraction techniques arise in the scientific field, with ultrasound-assisted extraction (UAE) constituting one of the most promising. Compared to other extraction methods, UAE requires less time and energy, leading to high extraction yields, while the quality of the extract is maintained [18]. The mechanism uses sound waves that by acoustic cavitation promote disruption in the tissues and, consequently, high compounds release [18]. However, some parameters, such as time, frequency, power, temperature, solvent, and ratio, influence the efficiency and yield of the bioactive compound extraction [19]. In this sense, it is imperative to apply statistic and computational methods, such as the response surface methodology (RSM), to optimize the extraction parameters. RSM allows us to understand the interaction between the factors and the effects of different parameters, through equations that relate to responses and experimental parameters [20]. In the present work, a RSM was applied to obtain the optimal extract from *A. arguta* leaves using UAE as green extraction technology. The effects of the solid:liquid ratio, time, and intensity on the antioxidant/antiradical activity (evaluated by ABTS, DPPH and FRAP assays) were studied. Additionally, the content of phenolic compounds, as well as the radical scavenging capacity and the in vitro effect on skin cell lines were evaluated for the optimal extract.

## 2. Materials and Methods

### 2.1. Chemicals

Most of the reagents were acquired from Sigma-Aldrich (Steinheim, Germany). For HPLC analysis, the solvents employed were supplied by Merck (Darmstadt, Germany). A human immortalized non-tumorigenic keratinocyte cell line (HaCaT) was obtained from CLS Cell Lines Service (Germany), while human foreskin fibroblasts (HFF-1) were provided from the American Type Culture Collection (ATCC Number: SCRC-1041; ATCC, Manassas, VA, USA). Cells reagents were supplied by Invitrogen Corporation (Life Technologies, S.A., Madrid, Spain).

### 2.2. Samples

*Actinidia arguta* leaves were harvested randomly from 10 different species in October 2019 in Mini-Kiwi Farm (GPS: 41.376705, −8.471039). The identification was confirmed by a botanist member of the research team and a voucher (No. 2234) of the plant has been deposited in GRAQ—Instituto Superior de Engenharia do Porto, Portugal. The leaves were dehydrated (Excalibur Food Dehydrator, Sacramento, CA, USA) at 41 °C for 24 h and grinded in a miller (Moulinex A320) to obtain particles with a mean size of 1 mm. Afterwards, samples were stored at 4 °C until extraction.

### 2.3. Ultrasound-Assisted Extraction

The UAE was carried out in an ultrasonic probe processor (Sonic Vibracell, model VCX50, Newtown, CT, USA) associated with a probe tip No. 630-0219 with 13 mm of diameter. Water was used as an extractor solvent and the experiments were carried out according to the RSM design (Section 2.4). After ultrasonic extraction, the extracts were filtered through Whatman n° 1 paper and frozen at −80 °C for subsequent lyophilization (Telstar, model Cryodos–80, Barcelona, Spain). Then, samples were stored at room temperature until further analysis.

### 2.4. Experimental Design and Optimization

The experimental design using the RSM was applied to optimize the antioxidant extraction from kiwiberry leaves through UAE, aiming to maximize the total phenolic content (TPC) and the antioxidant/antiradical activity. For this purpose, a Box–Behnken design (BBD) with five central points was employed to determine the optimal extraction conditions. The three independent variables under analysis were solid:liquid ratio (*X*_1_, % *w*/*v*), ultrasonic time (*X*_2_, min) and ultrasonic intensity (*X*_3_, W/m^2^). The solid:liquid ratio ranged between 2.5 and 10% *w*/*v*, while the ultrasonic time varied from 20 to 60 min and the ultrasonic intensity ranged from 30 to 70 W/m^2^. The responses studied were TPC (*Y*_1_, mg of gallic acid equivalents (GAE)/g of extract on dry weight (dw)), ABTS (*Y*_2_, IC_50_ µg/mL), DPPH (*Y*_3_, IC_50_ µg/mL) and FRAP (*Y*_4_, µmol of ferrous sulphate equivalents (FSE)/g dw). A total of 17 experiments were randomly performed (Table 1). The results were statistically analyzed through software Design Expert Version 11 (Stat-Ease Inc., Minneapolis, MN, USA) with the intention of predicting the model fitting and ascertaining the optimal extraction conditions based on the desirability function combined response surfaces, contour plots and predictive regression equations. A denoting significance of *p* < 0.05 was accepted. Afterwards, a new set of experiments was carried out under the critical optimal values to appraise the accuracy of the model. The experimental values obtained under the optimal extraction conditions were compared with those predicted by the model using a *t*-test.

#### 2.4.1. Total Phenolic Content

The TPC was evaluated spectrophotometrically according to the Folin–Ciocalteu procedure [21], with minor modifications [22]. Gallic acid was used as standard for the calibration (curve linearity range = 5–100 μg/mL; *R*^2^ > 0.998). The results were expressed as mg GAE/g dw.

#### 2.4.2. ABTS Radical Scavenging Activity Assay

The ABTS radical scavenging assay was conducted as described by Re et al. [23], with minor modifications. Ascorbic acid was used as the standard for the calibration curve (curve linearity range = 5–100 μg/mL; *R*^2^ > 0.996). Results were expressed as IC_50_ (μg/mL).

#### 2.4.3. DPPH Free Radical Scavenging Assay

The DPPH free radical scavenging assay was executed following the procedure described by Barros et al. [24], with minor amendments. The standard used for the calibration curve was Trolox (curve linearity range = 5–125 μg/mL; *R*^2^ > 0.991). The results were presented in terms of IC_50_ (μg/mL).

#### 2.4.4. Ferric Reducing Antioxidant Power Assay

The ferric reducing antioxidant power (FRAP) was evaluated according to Benzie and Strain [25], with minor alterations. The calibration curve was obtained with a solution of ferrous sulphate (FeSO_4_·7H_2_O) (curve linearity range = 25–500 μM; *R*^2^ > 0.999). The results were expressed in μmol FSE/g dw.

### 2.5. HPLC-PDA Analysis

The phenolic composition was identified and quantified by HPLC with photodiode array (PDA) detection, according to the procedure described by Moreira et al. [26]. A Gemini C_18_ column (250 mm × 4.6 mm, 5 μm) was used as stationary phase at 25 °C. Methanol (A) and water (B), both with 0.1% formic acid, were used as the mobile phase at a flow rate of 1.0 mL/min. The calibration curves (1–200 mg/L) were prepared with stock standards diluted with a mixture of methanol:water (50:50, *v*/*v*). The compounds were quantified at 280 nm, 320 nm and 360 nm, depending on the maximum absorption. The results were expressed as mg of each phenolic compound per gram of extract on dw (mg/100 g dw).

### 2.6. Evaluation of In Vitro Scavenging Capacity of Reactive Oxygen Species

The determination of the reactive oxygen species (ROS) scavenging capacity was carried out using a Synergy HT Microplate Reader (BioTek Instruments, Inc., Winooski, VT, USA). Gallic acid and catechin were used as positive controls. The optimal extract and the positive controls were dissolved in the phosphate buffer for each assay.

#### 2.6.1. Superoxide Anion Radical Scavenging Assay

The superoxide anion radical (O_2_^●−^) quenching capacity of the optimal extract from kiwiberry leaves was performed according to Gomes et al. [27]. The reaction mixture occurred by the addition of β-Nicotinamide adenine dinucleotide (NADH) (166 μM), Nitrotetrazolium blue chloride (NBT) (43 μM), the optimal extract at different concentrations, and Phenazine methosulfate (PMS) (2.7 μM), being these solutions previously dissolved in 19 mM phosphate buffer, pH 7.4. The absorbance was measured at 560 nm and 37 °C during 5 min. The results were expressed in terms of IC_50_ (μg/mL) of the NBT reduction to diformazan.

#### 2.6.2. Hypochlorous Acid Scavenging Assay

The hypochlorous acid (HOCl) scavenging capacity of the optimal extract and positive controls was evaluated following the methodology described by Gomes et al. [27]. A HOCl solution was prepared through 1% (*m*/*v*) NaOCl solution with the pH of 6.2, adjusting with 10% H_2_SO_4_. The reaction mixture was formed by 100 mM phosphate-buffered solution at pH 7.4, the optimal extract at different concentrations, of dihydrorhodamine 123 (DHR) (5 μM) and HOCl (5 μM). This fluorimetric assay was performed at 37 °C and at the emission wavelength of 528 nm, with excitation at 485 nm. The results were expressed as the inhibition, in IC_50_, of HOCl-induced oxidation of DHR.

### 2.7. Evaluation of In Vitro Cell Effects

The cell viability was screened in skin (immortalized human keratinocytes and fibroblasts, HaCaT and HFF-1, respectively) cell lines through a 3-(4,5-dimethylthiazol-2-yl)-2,5-diphenyltetrazolium bromide (MTT) assay, according to Pinto et al. [28]. Passages 83–84 and 11–12 from HaCaT and HFF-1, respectively, were exposed to different concentrations (0.1–1000 µg/mL) of the optimal extract. Positive (DMEM) and negative controls (1% (*w*/*v*) Triton X-100) were used. The absorbance was read at 590 nm, with a background subtraction at 630 nm. The results were presented in percentages of cell viability (% cell viability).

### 2.8. Statistical Analysis

All assays were performed in triplicate and the results were expressed as mean ± standard deviation. The statistical analysis was performed using a one-way analysis of variance (ANOVA) followed by Tukey’s HSD test with *p* < 0.05, through IBM SPSS Statistics 27.0 software (SPSS Inc., Chicago, IL, USA).

## 3. Results

### 3.1. Optimization of UAE

RSM constitutes a useful mathematical tool to optimize complex variables and reduce the number of experiments needed to determine the optimal extraction points [29]. In this study, RSM was applied to achieve the best extraction condition using UAE and considering different independent variables, namely time, solid:liquid ratio and intensity. The responses studied were TPC and antioxidant/antiradical activity evaluated by ABTS, DPPH and FRAP assays. As reported in Table 1, 17 experimental points were randomly run. The predicted and experimental values of TPC, FRAP, DPPH and ABTS assays are summarized in Table 1.

The TPC values ranged between 60.27 mg GAE/g dw (Run 15; 6.25% *w*/*v*, 60 min, 30 W/m^2^) and 117.22 mg GAE/g dw (Run 1; 10% *w*/*v*, 40 min, 30 W/m^2^). These results are in line with the ones obtained by Silva et al. for kiwiberry leaves extracted by subcritical water extraction (SWE) (68.78–109.72 mg GAE/g dw) [9]. Nevertheless, Silva et al. reported higher results for the kiwiberry leaf extracts obtained by microwave-assisted extraction (MAE) (120.99–629.48 mg GAE/g dw) [10]. Likewise, Almeida et al. achieved a higher TPC for the kiwiberry leaf extracts prepared by maceration during 1 h at 50 °C (140.72–440.71 mg GAE/g dw) [8]. In the same line, kiwiberry leaves extracted by multi-frequency multimode modulated (MMM) technology showed a substantially higher TPC (246.68 mg GAE/g dw) [7]. Comparing the values obtained in the present study with the values reported by different authors for the same by-product, it is possible to conclude that the solvent used (ethanol) or the temperature and time employed can influence the extraction [10,30]. In the present study, the temperature effect was not screened.

Regarding the antioxidant/antiradical activities, the IC_50_ values of ABTS varied from 265.02 µg/mL (Run 10; 6.25% *w*/*v*, 40 min, 50 W/m^2^) to 728.13 µg/mL (Run 17; 6.25% *w*/*v*, 60 min, 70 W/m^2^), being in accordance with the different extracts obtained by SWE (313.20–530.40 µg/mL) [9]. However, the MAE extracts exhibited lower IC_50_ values (131.58–219.14 µg/mL) [10]. In the DPPH assay, the IC_50_ values varied from 304.02 µg/mL (Run 1; 10% *w*/*v*, 40 min, 30 W/m^2^) to 978.72 µg/mL (Run 10; 6.25% *w*/*v*, 40 min, 50 W/m^2^), being in line with the values reported by Silva et al. for kiwiberry leaves extracted by SWE (497.10–625.60 µg/mL) [9]. Nevertheless, the values achieved in the present study were higher than the ones described by Silva et al. (95.22–211.14 µg/mL) [10] and Marangi et al. (270.17 µg/mL) [7] for MAE and MMM extracts, respectively. In addition, Almeida et al. achieved IC_50_ values of 53.95 µg/mL and 1097.28 µg/mL, respectively, for the alcoholic and hydroalcoholic extracts obtained by maceration [8]. Considering the FRAP assay, the results ranged between 584.88 µmol FSE/g dw (Run 4; 6.25% *w*/*v*, 40 min, 50 W/m^2^) and 1440.13 µmol FSE/g dw (Run 1; 10% *w*/*v*, 40 min, 30 W/m^2^). As occurred in other assays, the kiwiberry leaf extracts prepared by SWE presented similar results to this work (655.91–941.43 µmol FSE/g dw) [9]. However, the UAE extract presented better values in Run 1 (10% *w*/*v*, 40 min, 30 W/m^2^), Run 3 (10% *w*/*v*, 40 min, 70 W/m^2^) and Run 7 (10% *w*/*v*, 20 min, 50 W/m^2^) than the ones described by Silva et al. [9]. Oppositely, Silva et al. [10], Almeida et al. [8] and Marangi et al. [7] obtained higher results for this assay. As mentioned above, these authors used high temperatures (50, 72 and 94 °C) and ethanol as the solvent extractor [8,10]. However, in terms of economic and environmental impact, the UAE technique is very promissory, employing shorter extraction times, reduced amounts of solvents and energy [31]. In addition, UAE can be scaled-up into a pilot scale [32].

The mathematical model for the response variables was represented using quadratic functions, which were deducted by the analysis of variance (ANOVA) applying a Fisher’s *F*-test, as shown in Table 2. The quadratic model equations represent the correlations between dependent variables, including TPC (*Y*_1_), ABTS (*Y*_2_), DPPH (*Y*_3_) and FRAP (*Y*_4_), and the independent variables of the solid:liquid ratio (*X*_1_), time (*X*_2_) and intensity (*X*_3_) determined by multiple regression analysis of experimental data as follows below:

(1) $$Y_{1}=69.33+9.80X_{1}-6.08X_{2}+2.06X_{3}-11.01X_{1}.X_{2}-6.64X_{1}.X_{3}+0.4382X_{2}.X_{3}+20.96{X_{1}}^{2}-10.46{X_{2}}^{2}+8.72{X_{3}}^{2}$$

(2) $$Y_{2}=503.37-94.81X_{1}+25.25X_{2}+13.35X_{3}+49.86X_{1}.X_{2}+7.59X_{1}.X_{3}+72.46X_{2}.X_{3}-98.82{X_{1}}^{2}+101.50{X_{2}}^{2}-22.83{X_{3}}^{2}$$

(3) $$Y_{3}=802.61-95.38X_{1}-17.82X_{2}+4.73X_{3}+130.03X_{1}.X_{2}+33.87X_{1}.X_{3}-26.70X_{2}.X_{3}-156.10{X_{1}}^{2}-0.2687{X_{2}}^{2}-146.69{X_{3}}^{2}$$

(4) $$Y_{4}=725.32+191.77X_{1}-27.66X_{2}-2.96X_{3}-97.58X_{1}.X_{2}-52.47X_{1}.X_{3}-31.54X_{2}.X_{3}+255.03{X_{1}}^{2}-104.93{X_{2}}^{2}+112.23{X_{3}}^{2}$$

where *Y* represents the dependent variables (TPC, *Y*_1_; ABTS, *Y*_2_; DPPH, *Y*_3_; FRAP, *Y*_4_), and *X*_1_, *X*_2_ and *X*_3_ are the coded independent variables for solid:liquid ratio, time and intensity, respectively.

According to Table 2 and the equations above, the independent variable *X*_1_ (solid: liquid ratio; % *w*/*v*) had a significant influence (*p* < 0.05) on *Y*_1_, *Y*_2_ and *Y*_4_ responses (TPC, ABTS and FRAP, respectively). On the other hand, the independent variable *X*_2_ (time; min) showed a significant effect (*p* = 0.0272) on the TPC response (*Y*_1_). The quadratic term for *X*_1_ exhibited a significant effect (*p* < 0.01) on *Y*_1_ and *Y*_4_ responses (TPC and FRAP, respectively). However, the quadratic terms of *X*_2_ and *X*_3_ and the *X*_1_*.X*_2_ interaction demonstrated a significant influence (*p* < 0.05 and *p* < 0.01, respectively) on the TPC response. Considering the equations presented above, the negative impact of time and respective quadratic term pointed out a negative influence on TPC results, while the solid:liquid ratio exhibited a positive effect, and the ultrasonic intensity had no significant impact. Additionally, the solid:liquid ratio displayed a substantially negative effect on the ABTS response, while the DPPH results were not influenced by the independent variables studied. Likewise, the antioxidant results by the FRAP assay were positively influenced by an increase in the solid:liquid ratio. The *lack of fit* for all responses was found to not be significant (*p* > 0.2736) and the *ratio* was higher than 4 (as desirable), which support an adequate model fitting and signal-to-noise ratio. Regarding the *R*^2^ value, TPC showed a high *R*^2^ value and adjusted *R*^2^ (0.942 and 0.867, respectively), underlining a good adequacy of the model to this dependent variable. On the same hand, the *R*^2^ value for the FRAP response was 0.886 and the adjusted *R*^2^ was 0.739. Besides the low adjusted *R*^2^ values obtained for ABTS and DPPH responses, the experimental model was successfully validated based on the non-significance (*p* > 0.05) of the *lack of fit* and signal-to-noise ratios above 4 for all responses studied, as well as considering the high adjusted *R*^2^ values achieved for TPC and FRAP responses. It is noteworthy that significant *p* values were accomplished for the model applied to TPC and FRAP variables with *p* = 0.0015 and *p* = 0.0136, respectively, which denoted a good model fitting to these responses and reinforced the validation and effectiveness of the experimental design applied to the recovery of bioactive compounds from kiwiberry leaves. The justification for these observed responses may be justified by the different methodology principles of spectrophotometric methods used (TPC and FRAP) that are based on oxidation/reducing reactions and single electron transfer mechanism [33].

### 3.2. Response Surface Analysis

**Figure 1:**
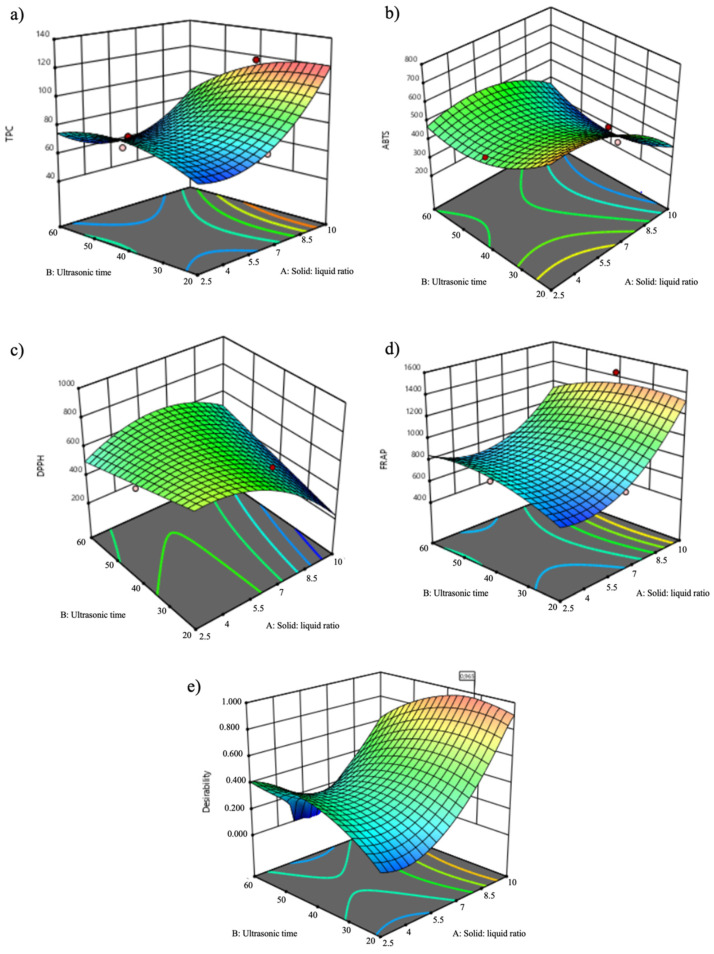
Response surface plots for interaction effects of solid:liquid ratio (% *w*/*v*) and time (min) on TPC (**a**), ABTS (**b**), DPPH (**c**) and FRAP (**d**) extraction and on the desirability index for combined responses of UAE and kiwiberry leaf extracts at fixed intensity (30 W/m^2^) (**e**).

The relationship between the independent (solid: liquid ratio, time, and intensity) and the dependent (TPC, ABTS, DPPH and FRAP assays) variables was illustrated in tridimensional (3D) representation of the response surface contour plots generated by the model for the extraction of bioactive compounds from kiwiberry leaves at the fixed intensity of 30 W/m^2^ (Figure 1).

As depicted in Figure 1, the response surfaces of TPC and FRAP assays displayed a similar behavior, while ABTS and DPPH showed distinct response profiles. Concerning TPC, the solid:liquid ratio was the main influencing factor, presenting a substantial increase at the highest solid:liquid ratio tested of 10% (*w*/*v*), as shown in Figure 1a. Time also exerted a considerable influence on TPC results, with the best outcomes being determined at intermediate extraction times (30–40 min) and the lowest results at lower and higher times. This response profile corroborates what is depicted in Figure 1e with a similar profile achieved in the desirability graph. At 2.5% (*w*/*v*), 20 min and 50 W/m^2^, the TPC response was probably related with the isolation of some bioactive compounds with antioxidant properties. For solid:liquid ratios higher than 2.5% (*w*/*v*), a steady rise was observed until reaching the highest values at 10% (*w*/*v*), which was further attested as the optimal solid:liquid ratio. Likewise, the FRAP results were efficiently maximized with a solid:liquid ratio of 10% (*w*/*v*), while time had an insignificant effect (Figure 1d). Besides the different response surfaces, ABTS and DPPH responses showed better results at the highest solid:liquid ratios owing to the lower IC_50_ values that underline the higher antiradical potential and a positive influence of this variable on antiradical activity assays. Considering all the results, the solid:liquid ratio was the major influencing variable on the extraction of kiwiberry leaves’ antioxidant compounds, with a remarkable impact on TPC, ABTS and FRAP responses, followed by time that encompasses a substantial effect on TPC results. Nevertheless, the impact of intensity was negligible on all responses studied.

According to the desirability graph (Figure 1e), the predicted optimal UAE conditions were carried out with a solid:liquid ratio of 10% (*w*/*v*), for 31.11 min and an ultrasonic intensity of 30 W/m^2^ (*R*^2^ = 0.965). As shown in Table 3, the predicted results obtained by the CCD were similar to the experimental values of the optimal extract, without significant differences (*p* > 0.05). These results support that the model is well suited for the extraction of antioxidant/antiradical compounds from kiwiberry leaves under the optimal UAE conditions. Therefore, the designed model is good for predicting the optimal extraction conditions.

The present study emphasizes the potential of UAE as a proficient and eco-friendly extraction technology to isolate bioactive compounds endowed with potent antioxidant properties from kiwiberry leaves employing green extraction solvents (namely water), short times (31.11 min) and low energy consumption (30 W/m^2^). Furthermore, UAE has great potential for up scaling and implementation at the industrial level, owing to its elevated cost–benefit ratio allied to few environmental impacts. Notably, the promising outcomes achieved in the previous assays may be attributed to the phenolic composition, particularly rich in neochlorogenic and chlorogenic acids, caffeoylquinic acid derivatives, catechin, epicatechin, kaempferol and quercetin derivatives (Table 4).

### 3.3. Characterization of the Optimal Extract

#### 3.3.1. Phenolic Profile Identification and Quantification by HPLC-PDA

**Figure 2:**
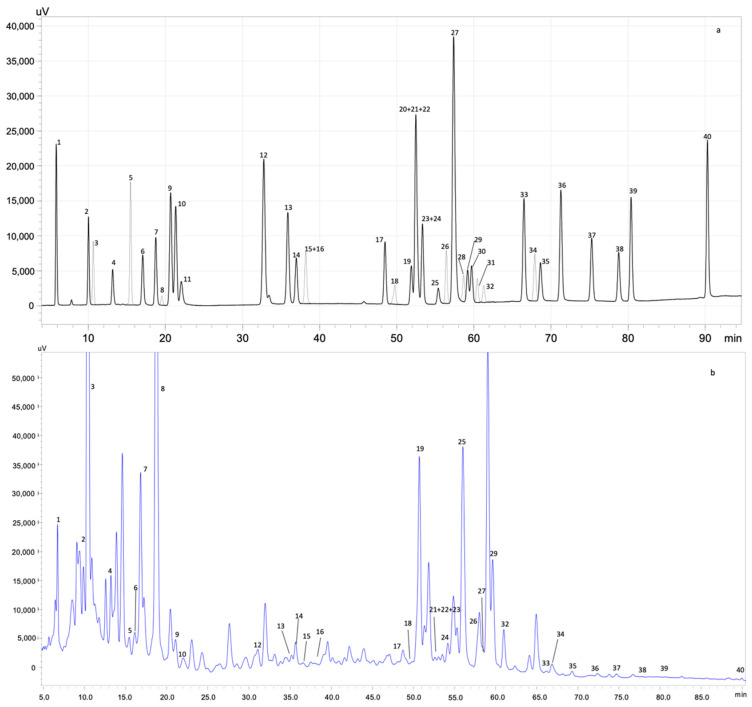
HPLC-PDA chromatogram monitored at 280 nm for (**a**) polyphenol standard mixture of 5 mg/L and (**b**) optimal extract from kiwiberry leaves; peak identification: (1) gallic acid, (2) protocatechuic acid, (3) neochlorogenic acid, (4) (+)-catechin, (5) caftaric acid, (6) caffeine, (7) chlorogenic acid, (8) 4-*O*-caffeyolquinic acid, (9) vanillic acid, (10) caffeic acid, (11) syringic acid, (12) (−)-epicatechin, (13) *p*-coumaric acid, (14) ferulic acid, (15) sinapic acid, (16) trans-polydatin, (17) naringin, (18) 3,5-di-caffeoylquinic acid, (19) quercetin-3-*O*-galactoside, (20) resveratrol, (21) quercetin-3-*O*-glucopyranoside, (22) rutin, (23) phloridzin, (24) ellagic acid, (25) 3,4-di-*O*-caffeoylquinic acid, (26) myricetin, (27) cinnamic acid, (28) quercitrin, (29) kaempferol-3-*O*-glucoside, (30) isorhamnetin-3-*O*-glucoside, (31) kaempferol-3-*O*-rutinoside, (32) isorhamnetin-3-*O*-rutinoside, (33) naringenin, (34) trans-epsilon viniferin, (35) quercetin, (36) phloretin, (37) tiliroside, (38) kaempferol, (39) apigenin and (40) chrysin.

The identification and quantification of the phenolic compounds present in the optimal extract were carried out by HPLC-PDA (Table 4). Figure 2 presents the HPLC-PDA chromatograms obtained for the mixture of phenolic standards and the optimal extract.

**Figure 3:**
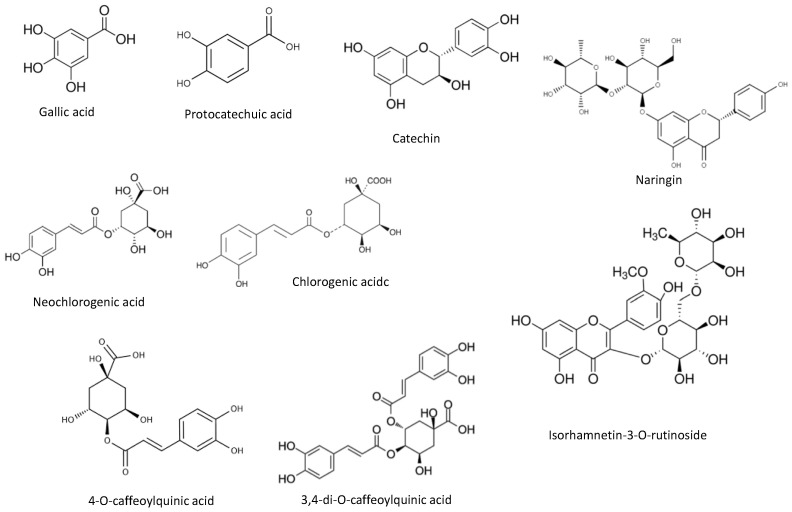
Chemical structures of the principal compounds identified and quantified through HPLC-PDA analysis in the optimal extract from kiwiberry leaves.

As reported in Table 4, 28 phenolic compounds were identified and quantified in the optimal kiwiberry leaf extract. The principal class of compounds was phenolic acid (2103 mg/100 g dw), representing 82% of the total phenolic content. Neochlorogenic acid was the principal phenolic acid, followed by 3,4-di-*O*-caffeoylquinic acid and chlorogenic acid (761, 491 and 196 mg/100 g dw, respectively). Flavonols were the second major class of compounds, being responsible for 7.9% of the total phenolic composition. Kaempferol-3-*O*-glucoside (27.6 mg/100 g dw) and isorhamnetin-3-*O*-rutinoside (103 mg/100 g dw) were the two main flavonols quantified. Regarding flavanols, epicatechin (20.2 mg/100 g dw) and catechin (80.9 mg/100 g dw) were identified. Caffeine was also present in the optimal extract of kiwiberry leaves. Figure 3 represents the main phenolic compounds identified and quantified in the optimal extract from kiwiberry leaves. These results were significantly higher than the ones reported by Silva et al. for SWE extracts [9]. The main phenolic compounds identified in SWE extracts were phenolic acids (in the extract obtained at 160 °C; 1842.1 mg/100 g dw), being gallic and protocatechuic acids the principal compounds quantified [9]. Furthermore, flavonols and flavanols were detected in the SWE extracts [9]. Almeida et al. also quantified phenolic compounds in the aqueous, hydroalcoholic and alcoholic extracts of kiwiberry leaves obtained by maceration [8]. However, the total amount of the phenolic compounds varied between 108.07 and 238.76 µg/mg dw) [8], being lower than the ones achieved in the present study.

The phenolic profile is very promissory regarding skin application, since the neochlorogenic acid, the principal compound quantified, has skin photoaging and hydration effects [34]. On the other hand, caffeoylquinic acids present an inhibitory effect against tyrosinase, inhibiting melanogenesis and preventing the hyperpigmentation signals [35,36], while catechin already proved the ability to prevent aging signals and prevent the negative effects of ultraviolet radiation [37].

#### 3.3.2. In Vitro Scavenging Capacity of ROS

ROS are generated in mitochondria as a consequence of the cellular metabolism [38]. The first barrier against the oxidative stress generated by these species is the non-enzymatic antioxidants, such as phenolic compounds [39]. However, when the ROS production exceeds the antioxidant capacity of the cellular systems, the oxidative stress generated may led to cell damage [38]. Table 5 summarizes the radical scavenging capacity of the optimal extract. As it is possible to observe, the optimal extract of kiwiberry leaves presented a significant capacity to scavenge all tested ROS. Concerning the scavenging capacity of O_2_^●−^, the IC_50_ of the optimal extract was 220.13 μg/mL, while gallic acid showed the best value and catechin the worst (IC_50_ = 52.49 and 590.18 μg/mL, respectively). Significant differences (*p* < 0.05) were observed between all samples. Silva et al. evaluated the in vitro radicals scavenging activity of kiwiberry leaf extracts obtained by SWE [9]. Comparing to the present study, the authors achieved a considerably lower quenching capacity of O_2_^●−^ (IC_50_ ranged from 321.6 μg/mL to 539.7 μg/mL) [9]. Nonetheless, in another study, Silva et al. extracted kiwiberry leaves with MAE and achieved an IC_50_ = 61.50 μg/mL for the aqueous extract [10]. Concerning the extraction technique employed, Eddine et al. screened the in vitro antioxidant activity of *Rumex vesicarius* leaves using UAE, Soxhlet and conventional extractions [40]. The results obtained demonstrated that the UAE extract presented an IC_50_ similar to the one reported in Table 5 for the optimal extract (IC_50_ = 264.56 μg/mL) [40].

Furthermore, the optimal extract exhibited a good scavenging of HOCl (IC_50_ = 10.26 μg/mL). However, the positive controls presented the highest HOCl quenching potential, with significant differences between the optimal extract and the positive controls (*p* < 0.05). Compared with other studies, the optimal extract displayed a substantially higher HOCl scavenging capacity than the kiwiberry leaf extract obtained by SWE [9].

As previously reported by Kitagawa et al., chlorogenic acid is an example of a phenolic acid that may neutralize ROS and, consequently, prevents the ultraviolet-B erythema formation [41].

#### 3.3.3. In Vitro Cell Studies

**Figure 4:**
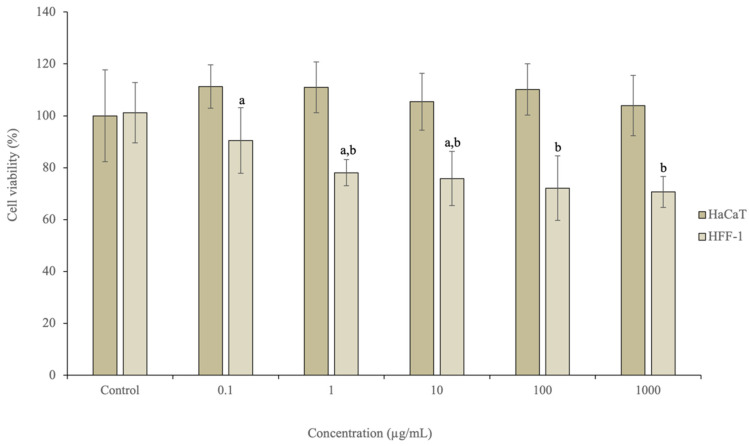
Effects of the optimal extract exposure on the viability of HaCaT and HFF-1 cell lines at different concentrations (0.1–1000 µg/mL), measured by the MTT assay (*n* = 3). Different letters (a, b) mean significant differences between the different tested concentrations (*p* < 0.05).

The in vitro assays are a good methodology to screen the effect of bioactive compounds in living cells, being fast, not too expensive and reproducible [42]. The MTT (3-[4,5-dimethylthiazol-2-yl]-2,5-diphenyltetrazolium bromide) assay is a colorimetric methodology based on the conversion of the soluble tetrazolium salt into an insoluble purple formazan [43]. The safety of the optimal extract of kiwiberry leaves was assessed on skin cell lines (HaCaT and HFF-1). Figure 4 summarizes the results obtained after exposure to the optimal extract. As it is possible to observe, the optimal extract did not affect the HaCaT viability, presenting results around 100% for all tested concentrations without significant differences (*p* > 0.05). Regarding HFF-1, the viability ranged between 90.50% and 70.63%, respectively, for the concentration of 0.1 µg/mL and 1000 µg/mL. The statistical analysis revealed differences (*p* < 0.006) between the highest (100 and 1000 µg/mL) and the lowest (0.1 µg/mL) concentrations tested.

Marangi et al. evaluated the potential cytotoxicity effects of kiwiberry leaf extracts obtained by MMM on HaCaT and HFF-1 cells [7]. The authors tested different concentrations (15.63 μg/mL–500 μg/mL) and verified that the extracts did not lead to a decrease in the cellular viability of HaCaT, while in HFF-1 a viability of 66% at the highest concentration tested (500 μg/mL) was reported [7]. Considering the results achieved and the skin structure, these results emphasize the good potential of the kiwiberry leaf extracts for application in dermatological products.

## 4. Conclusions

In the present study, an optimized and efficient UAE method was employed to obtain several classes of polyphenols with antioxidant activity from kiwiberry leaves. Three independent variables (solid:liquid ratio, ultrasonic time, and ultrasonic intensity) were optimized by a Box–Behnken design. The optimal extraction conditions were achieved using a solid:liquid ratio of 10% (*w*/*v*), for 31.11 min and under an amplitude of 30 W/m^2^. The kiwiberry leaves’ optimal extract revealed a great variety of phenolic compounds, particularly neochlorogenic and chlorogenic acids, caffeoylquinic acid, catechin, kaempferol-3-*O*-glucoside and isorhamnetin-3-*O*-rutinoside. In addition, a good scavenging efficiency was observed for O_2_^●−^ and HOCl. The in vitro cell results demonstrated the absence of toxic effects of the optimal extract on keratinocytes and fibroblasts, highlighting the skin cell lines compatibility. This work reinforces the potential of UAE as a green, effective and sustainable extraction technique to recover bioactive compounds with antioxidant capacity from kiwiberry leaves, allowing, in future, its use at the cosmetic industrial level. Further studies, such as the evaluation of the safety and toxicity of the extract in human volunteers, should be carried out.

## Figures and Tables

**Table 1:** Independent variables used for the central composite design (CCD) and predicted and experimental values of total phenolic compounds (TPC) (mg gallic acid equivalents (GAE)/g dw), ABTS (IC_50_ μg/mL), DPPH (IC_50_ μg/mL) and FRAP (µmol FSE/g dw) of kiwiberry leave extracts obtained by CCD.

	Independent Variables			Dependent Variables							
Point	Extraction Conditions			*Y*_1_, TPC (mg GAE/g dw)		*Y*_2_, ABTS (IC_50_, µg/mL)		*Y*_3_, DPPH (IC_50_, µg/mL)		*Y*_4_, FRAP (µmol FSE/g dw)	
Run	*X*_1_ (Solid: Liquid Ratio, % *w*/*v*)	*X*_2_ (t, min)	*X*_3_ (Intensity, W/m^2^)	Predicted	Experimental	Predicted	Experimental	Predicted	Experimental	Predicted	Experimental
1	10	40	30	113.39	117.22 ± 6.08	265.97	288.74 ± 3.18	365.83	304.02 ± 32.87	1339.78	1440.13 ± 63.43
2	2.5	40	70	97.91	94.07 ± 3.32	482.29	459.52 ± 8.35	566.06	627.87 ± 30.46	950.32	849.96 ± 48.09
3	10	40	70	104.22	108.56 ± 9.19	307.85	275.37 ± 7.78	443.04	469.99 ± 40.84	1228.93	1261.83 ± 34.60
4	6.25	40	50	69.33	69.83 ± 8.91	503.37	506.84 ± 40.24	802.61	669.04 ± 4.57	725.32	584.88 ± 47.81
5	10	60	50	72.55	67.45 ± 4.35	486.34	483.79 ± 55.63	663.07	785.75 ± 49.22	941.95	887.67 ± 90.80
6	6.25	20	70	75.29	74.03 ± 4.85	497.67	517.88 ± 32.68	704.90	765.77 ± 37.67	788.86	834.93 ± 61.66
7	10	20	50	106.73	103.66 ± 11.98	336.13	348.40 ± 7.97	438.66	350.84 ± 19.09	1192.43	1113.46 ± 96.87
8	6.25	40	50	69.33	78.41 ± 6.55	503.37	583.04 ± 14.37	802.61	759.46 ± 43.04	725.32	818.59 ± 64.82
9	6.25	40	50	69.33	68.86 ± 3.08	503.37	637.54 ± 72.35	802.61	691.25 ± 11.80	725.32	847.63 ± 81.65
10	6.25	40	50	69.33	64.04 ± 3.33	503.37	265.02 ± 5.35	802.61	978.72 ± 6.19	725.32	662.02 ± 90.97
11	6.25	40	50	69.33	65.49 ± 3.35	503.37	524.40 ± 36.11	802.61	914.57 ± 9.49	725.32	713.47 ± 84.80
12	2.5	60	50	74.95	78.02 ± 7.87	576.25	563.98 ± 33.54	593.76	681.58 ± 11.61	753.57	832.54 ± 75.51
13	2.5	20	50	65.11	70.20 ± 8.88	625.46	628.02 ± 71.38	889.47	766.80 ± 53.25	613.72	668.00 ± 92.03
14	2.5	40	30	80.50	76.17 ± 10.55	470.76	503.25 ± 64.32	624.32	597.37 ± 24.74	851.30	818.40 ± 62.44
15	6.25	60	30	59.00	60.27 ± 9.40	521.46	501.25 ± 3.94	659.79	598.92 ± 11.35	739.45	693.38 ± 64.38
16	6.25	20	30	72.05	71.29 ± 4.06	615.89	580.86 ± 21.28	642.03	791.67 ± 15.04	731.69	710.31 ± 64.18
17	6.25	60	70	64.00	64.76 ± 4.08	693.10	728.13 ± 38.94	615.85	466.22 ± 30.70	670.47	691.86 ± 66.16

**Table 2:** Model summary and analysis of variance (ANOVA) of TPC, ABTS, DPPH and FRAP of the kiwiberry leaf extracts.

Source	Sum of Squares				Mean Squares				*F* Value				*p*-Value			
	*Y*_1_	*Y*_2_	*Y*_3_	*Y*_4_	*Y*_1_	*Y*_2_	*Y*_3_	*Y*_4_	*Y*_1_	*Y*_2_	*Y*_3_	*Y*_4_	*Y*_1_	*Y*_2_	*Y*_3_	*Y*_4_
Model	4346.36	192,100	355,700	724,300	482.93	21,339.45	395,24.97	80,474.35	12.63	1.69	1.52	6.03	0.0015 *	0.2506	0.2983	0.0136 **
*X*_1_. % *w*/*v*	768.69	71,906.90	72,773.42	294,200	768.69	71,906.90	72,773.42	294,200	20.11	5.70	2.79	22.06	0.0029 *	0.0484 **	0.1387	0.0022 *
*X*_2_. min	296.11	5100.15	2541.75	6119.17	296.11	5100.15	2541.75	6119.17	7.75	0.4041	0.0975	0.4588	0.0272 **	0.5452	0.7639	0.5199
*X*_3_. W/m^2^	33.93	1426.03	179.32	69.86	33.93	1426.03	179.32	69.86	0.8876	0.1130	0.0069	0.0052	0.3775	0.7466	0.9362	0.9443
*X*_1_.*X*_2_	484.48	9942.49	67,633.11	38,088.79	484.48	9942.49	67,633.11	38,088.79	12.67	0.7878	2.59	2.86	0.0092 *	0.4042	0.1513	0.1349
*X*_1_.*X*_3_	176.50	230.45	4588.16	11,010.93	176.50	230.45	4,588.16	11,010.93	4.62	0.0183	0.1760	0.8256	0.0687	0.8963	0.6874	0.3938
*X*_2_.*X*_3_	0.7683	21,004.62	2851.78	3978.14	0.7683	21,004.62	2,851.78	3978.14	0.0201	1.66	0.1094	0.2983	0.8913	0.2380	0.7505	0.6019
*X*_1_^2^	1850.28	41,114.03	102,600	273,900	1850.28	41,114.03	102,600	273,900	48.40	3.26	3.94	20.53	0.0002 *	0.1141	0.0877	0.0027 *
*X*_2_^2^	460.39	43,374.07	0.3040	46,360.86	460.39	43,374.07	0.3040	46,360.86	12.04	3.44	0.0000	3.48	0.0104 **	0.1062	0.9974	0.1045
*X*_3_^2^	319.80	2194.89	90,608.08	53,035.74	319.80	2194.89	90,608.08	53,035.74	8.37	0.1739	3.48	3.98	0.0232 **	0.6892	0.1045	0.0864
Residual	267.61	88,347.51	182,500	93,361.93	38.23	12,621.07	26,065.91	13,337.42								
*Lack of fit*	141.97	6733.69	106,800	45,833.46	47.32	2244.56	35,602.49	15,277.82	1.51	0.1100	1.88	1.29	0.3415	0.9498	0.2736	0.3934
Pure error	125.64	81,613.82	75,653.91	475,28.47	31.41	20,403.45	18,913.48	11,882.12								
Total	4613.97	280,400	538,200	817,600												
R2 *pred* (Y1)—0.4651					R2 *adjust* (Y1)—0.8674				*Ratio*—11.47							
R2 *pred* (Y2)—0.1610					R2 *adjust* (Y2)—0.2798				*Ratio*—4.96							
R2 *pred* (Y3)—-2.3950					R2 *adjust* (Y3)—0.2251				*Ratio*—4.23							
R2 *pred* (Y4)—0.0123					R2 *adjust* (Y4)—0.7390				*Ratio*—8.20							

* significance at *p* < 0.01; ** significance at *p* < 0.05.

**Table 3:** TPC and antioxidant/antiradical activity evaluated by ABTS, DPPH and FRAP assays of the optimal extract of kiwiberry leaves (10% *w*/*v*; 31.11 min; 30 W/m^2^).

	TPC (mg GAE/g dw)	ABTS (IC_50_; µg/mL)	DPPH (IC_50_; µg/mL)	FRAP (µmol FSE/g dw)
Experimental value	97.50 ± 2.74	249.46 ± 20.89	547.34 ± 21.44	1154.10 ± 85.85
Predicted value	119.12	284.85	304.05	1360.69
*p*	0.053	0.689	0.129	0.123

**Table 4:** Identification and quantification of the phenolic compounds and others present in the optimal extract from kiwiberry leaves through HPLC-PDA analysis. Results are expressed as mean ± standard deviations (mg of phenolic compound/100 g dw).

Compounds	(mg/ 100 g dw)
**Phenolic acids**	
Gallic acid	91.9 ± 4.6
Protocatechuic acid	174 ± 9
Neochlorogenic acid	761 ± 38
Caftaric acid	22.6 ± 1.1
Chlorogenic acid	196 ± 10
4-*O*-caffeoylquinic acid	338 ± 17
Vanillic acid	<LOD
Caffeic acid	<LOQ
Syringic acid	ND
*p*-coumaric acid	<LOD
Ferulic acid	4.13 ± 0.21
Sinapic acid	<LOQ
3,5-di-caffeoylquinic acid	7.86 ± 0.39
Ellagic acid	15.6 ± 0.8
3,4-di-*O*-caffeoylquinic acid	491 ± 25
Cinnamic acid	0.84 ± 0.04
**∑Phenolic acids**	**2103 ± 106**
**Flavanols**	
Catechin	80.9 ± 4.0
Epicatechin	20.2 ± 1.0
**∑Flavanols**	**101 ± 5**
**Flavanones**	
Naringin	64.3 ± 3.2
Naringenin	7.92 ± 0.40
**∑Flavanones**	**72.2 ± 3.6**
**Flavonols**	
Quercetin-3-*O*-galactoside	22.4 ± 1.1
Quercetin-3-*O*-glucopyranoside	7.08 ± 0.35
Rutin	9.18 ± 0.46
Myricetin	25.6 ± 1.28
Kaempferol-3-*O*-glucoside	27.6 ± 1.4
Isorhamnetin-3-*O*-glucoside	ND
Kaempferol-3-*O*-rutinoside	ND
Isorhamnetin-3-*O*-rutinoside	103 ± 5
Quercetin	4.96 ± 0.25
Tiliroside	0.85 ± 0.04
Kaempferol	2.79 ± 0.14
**∑Flavonols**	**203 ± 10**
**Flavones**	
Apigenin	<LOD
Chrysin	<LOQ
**∑ Flavones**	**–**
**Others**	
Caffeine	55.9 ± 2.8
*trans*-polydatin	2.11 ± 0.11
Resveratrol	<LOQ
Phloridzin	7.69 ± 0.38
*trans*-ε-viniferin	14.9 ± 0.7
Phloretin	<LOQ
**∑Others**	**80.6 ± 4.0**

ND: not detected; LOD: Limit of Detection; LOQ: Limit of Quantification.

**Table 5:** Superoxide anion radical (O_2_^●−^) and hypochlorous acid (HOCl) scavenging capacities of the optimal extract of kiwiberry leaves. Different letters (^a^, ^b^, ^c^) in the same column mean significant differences (*p* < 0.05) between samples.

Samples	ROS	
	O_2_^●−^	HOCl
	IC_50_ (µg/mL)	
Optimal extract	220.13 ± 3.41 ^b^	10.26 ± 0.35 ^b^
Positive controls		
Catechin	590.18 ± 14.31 ^c^	0.10 ± 0.01 ^a^
Gallic acid	52.49 ± 1.58 ^a^	0.60 ± 0.03 ^a^

## Data Availability

Data is contained within the article.
